# Electrophysiological Properties of Human Cortical Organoids: Current State of the Art and Future Directions

**DOI:** 10.3389/fnmol.2022.839366

**Published:** 2022-02-16

**Authors:** Clara Zourray, Manju A. Kurian, Serena Barral, Gabriele Lignani

**Affiliations:** ^1^Department of Clinical and Experimental Epilepsy, UCL Queen Square Institute of Neurology, London, United Kingdom; ^2^Developmental Neurosciences, Zayed Centre for Research Into Rare Disease in Children, GOS-Institute of Child Health, University College London, London, United Kingdom; ^3^Department of Pharmacology, UCL School of Pharmacy, London, United Kingdom; ^4^Department of Neurology, Great Ormond Street Hospital for Children, London, United Kingdom

**Keywords:** cortical organoids, electrophysiology, neurodevelopmental disorders, network activity, neuronal maturation

## Abstract

Human cortical development is an intricate process resulting in the generation of many interacting cell types and long-range connections to and from other brain regions. Human stem cell-derived cortical organoids are now becoming widely used to model human cortical development both in physiological and pathological conditions, as they offer the advantage of recapitulating human-specific aspects of corticogenesis that were previously inaccessible. Understanding the electrophysiological properties and functional maturation of neurons derived from human cortical organoids is key to ensure their physiological and pathological relevance. Here we review existing data on the electrophysiological properties of neurons in human cortical organoids, as well as recent advances in the complexity of cortical organoid modeling that have led to improvements in functional maturation at single neuron and neuronal network levels. Eventually, a more comprehensive and standardized electrophysiological characterization of these models will allow to better understand human neurophysiology, model diseases and test novel treatments.

## Introduction

The human cerebral cortex remains mostly inaccessible for direct functional investigation. Thus, the study of human cortical physiology and development largely depends on animal and, more recently, human induced pluripotent stem cells (hiPSCs)-derived models. Animal models, in particularly rodents, have provided a wealth of insight into cortical development and circuitry. However, some human-specific features of the cortex and its development, such as the presence of cortical folding, the developmental outer subventricular zone (oSVZ), human-specific cell types or the protracted development and migration of cortical interneurons, cannot be explored in rodent models (Chi et al., 1977; Hansen et al., 2010; Paredes et al., 2016; Boldog et al., 2018). These features can be critically relevant for some human diseases and the development of novel *in vitro* human models of the cortex has been a very active area of research in recent years (Pasça et al., 2015; Qian et al., 2016; Trujillo et al., 2019; Velasco et al., 2019). Although 2D hiPSC-derived cortical neuronal cultures align to human neural physiology to some extent at the single cell level, key aspects of the cortical physiological development are hampered such as signaling processes, neuronal migration, synapse formation and the emergence of a complex cortical network activity. Development of 3D neural cultures directed toward a dorsal forebrain fate, namely cortical organoids or cortical spheroids, provide a novel human-derived system to model several physiological aspects of human cortical development *in vitro* (Pasça et al., 2015; Qian et al., 2016; Paşca, 2018; Trujillo et al., 2019; Velasco et al., 2019). 3D cortical organoids have been recently used to study human cortical development and model diseases, enabling novel mechanistic insights into the human brain (Amin and Pasça, 2018). Although cortical organoids can be maintained for a very long time in culture, it remains unclear what level of functional maturation can be achieved. Importantly, mature cellular and network electrophysiological properties are required to develop relevant models for neurological disorders, especially those associated with abnormal patterns of activity such as epilepsy, early-onset neurodevelopmental disorders and psychiatric diseases. Here, we will review the electrophysiological properties of cortical organoids, both at the cellular and network level, and discuss recent advances to improve physiological relevance and maturation.

## Cellular and Network Electrophysiological Properties of Human Cortical Organoids

To date, several protocols have been developed to induce a dorsal forebrain fate in brain organoids and generate 3D cerebral cortex-like structures (Pasça et al., 2015; Qian et al., 2016; Trujillo et al., 2019; Velasco et al., 2019). However, only few have been comprehensively characterized using electrophysiology techniques (Table 1; Pasça et al., 2015; Trujillo et al., 2019).

**TABLE 1:** Electrophysiological properties of cortical organoid models.

References	Model	Base media	Cell types	Ephys organoid prep	Voltage-clamp		Current-clamp		Extracellular recordings		Multi-electrode array (MEA)	
								
					Methods	Results	Methods	Results	Methods	Results	Methods	Results
Pasça et al., 2015	Cortical spheroid (hCS)	Neurobasal A	Excitatory, inhibitory (few), astrocytes	Slices, in aCSF	K-gluconate internal solution. Spontaneous EPSCs recorded at −70 mV. Day 90–130.	Spontaneous EPSCs recorded in 86% of neurons and blocked by Kynurenic acid. Evoked EPScs also recorded after stimulation in slice.	K-gluconate internal solution. Day 90–130. Depolarizing current steps from 0 to 12 pA.	80% of neurons fired action potentials upon depolarizing current steps. Spontaneous and evoked action potentials also recorded.				
Sun et al., 2019	Cortical spheroid (hCS)	Neurobasal A	Excitatory, inhibitory (few), astrocytes	Wholemount, in aCSF			K-methanesulfonate internal (290 mOsm). 500 ms pulses from 0 to 35 pA in 5 pA increment, 4 s interval.	Max spike frequency WT = 18 Hz at 35 pA. Cm = 20 pF. RMP = −60 mV.				
Trujillo et al., 2019	Cortical organoid	Neurobasal	Excitatory, inhibitory (few), astrocytes	Plated on coated plates	K-gluconate internal solution (290 MOsm).	K + current and TTX-sensitive Na + currents recorded. Peak INa size = −1,466.86 pA. Peak IK size = 3,031.79 pA. Spontaneous EPSCs frequency at −60 mV = 0.25 Hz, amplitude = −19.92 pA. sEPSCs observed in 84% of neurons.	K-gluconate internal solution (290 MOsm).	TTX-sensitive train of AP recorded at 50 pA. Spontaneous AP firing frequency at −60 mV = 13.67 Hz			Cortical organoid plated per well of 12-well MEA plate containing 64 low-impedance (0.04 MOhm) platinum microelectrodes	Mean firing rate at 40 weeks = 18 Hz. Burst frequency at 10 months = 0.25 Hz
Negraes et al., 2021	Cortical organoid	Neurobasal	Excitatory, inhibitory (few), astrocytes								Cortical organoid plated per well of 12-well MEA plate containing 64 low-impedance (0.04 MOhm) platinum microelectrodes	Mean firing rate at 24 weeks = about 1.2 Hz
Birey et al., 2017	Cortico-subpallial assembloid	Neurobasal A	Excitatory, inhibitory, astrocytes	Slices, in aCSF	K-gluconate (low Cl-) internal solution used to distinguish between EPSCs and IPSCs, Cl- reversal = −91 mV, holding voltage = −40 mV	Spontaneous IPSCs and EPSCs recorded in migrated inhibitory (IPSCs = 20/mn; EPSCs = 15/mn) and excitatory cells (IPSCs = 18/mn; EPSCs = 10/mn). EPSCs blocked by kynurenic acid and IPSCs blocked by gabazine.	−5 pA to 5 pA current steps, step size not known. K-gluconate internal solution (290 MOsm).	Representative trace of train of action potentials in migrated inhibitory cell at 5 pA. Max AP firing of migrated eGFP + interneuron ≈ 7 Hz.				
Samarasinghe et al., 2021	Cortico-subpallial assembloid	DMEM-F12	Excitatory (CFuPNs, CPNs), inhibitory neurons, astrocytes, ORGs, RGCs, Cajal-Retzius cells	Wholemount, aCSF + 500 nM kainic acid					Patch pipette filled with aCSF. Field potentials digitized at 4,096 Hz.	Cx-GE = robust oscillatory activities at multiple frequencies over 5 mn period. Spectral density analysis shows multiple distinct oscillatory peaks ranging from 1–100 Hz. Cx-Cx = no measurable oscillatory activities. Cx-GE control: high gamma (65–80 Hz) = 13% total spectral power; Low gamma (35–45 Hz) = 15% total spectral power on average		
Miura et al., 2020	Cortico-striatal assembloid	Neurobasal A	hCS = excitatory, astrocytes: hStrS = inhibitory, excitatory, astrocytes, oligodendrocytes	Slices, in aCSF	K-gluconate internal solution (290 MOsm). oEPSCs recorded at −70 mV. 5-ms duration of 550-nm whole-field LED illumination. Cells recorded at 30°C.	After optogenetic stimulation of hCS, oEPSCs recorded in hStrS. 11/35 cells were responsive to opto stimulation (31.4%). Peak EPSC amplitude: −40 pA on average. sEPSC frequency in fused hStrS neurons fused (0.5 Hz) and unfused (0.1 Hz), significant increase.	K-gluconate internal solution (290 MOsm). oEPSPs holding voltage = −50 mV holding voltage; opto-evoked firing = −40 mV holding voltage. 5-ms duration of 550-nm whole-field LED illumination. Cells recorded at 30°C.	hStrS neuronal firing recorded following optogenetic stimulation of hCS. Recorded neurons in hStrS show increased spike frequency when fused to hCS (Max = 22 Hz average) compared to unfused (Max = 10 Hz average). RMP hCS = −60 mV; RMP hStrS = −80 mV. Spike amplitude = 75 mV. Spike threshold = −40 mV.				
Xiang et al., 2019	Cortico-thalamic assembloid	DMEM-F12/Neurobasal	hCOs = excitatory neurons, inhibitory neurons and astrocytes. hThO = mainly excitatory neurons, astrocytes	Slices, in aCSF			K-gluconate internal solution (290 MOsm). Thalamic neurons injected with hyperpolarizing and depolarizing current steps: from −10 to + 20 pA in 5 pA increments; 1 s steps from around −60 mV	10 of 21 neurons fired APs in non-fused hThOs, similar to 9 in 15 cells in fused. Increased firing frequency of hThO-derived neurons when fused (17 Hz at 20 pA) to COs compared to unfused (6 Hz at 20 pA).				
Fagerlund et al., 2021	Cerebral organoid with microglia	DMEM-F12/Neurobasal	Excitatory, inhibitory, astrocytes, retina, neural crest, microglia	Slices, in aCSF	Day 107–165. 350 μm slices. K-gluconate internal solution. Cells recorded at 23°C.	Max sEPSC amplitude around 40 pA. In cerebral organoids without microglia: 0 out of 14 cells with sEPSCs. In cerebral organoids with microglia: 5 out of 16 cells with sEPSCs. Increased K + and Na + current densities in cerebral organoids with microglia.	Day 107–165. 350 μm slices. K-gluconate internal solution. Cells recorded at 23°C.	Increase in proportion of cells able to fire trains of action potentials in organoids with microglia (21 out of 21) compared to organoids without microglia (8 out of 14).			Day 107–165. 500 μm slices placed in chamber of 3D-MEA. Recordings performed at 23°C.	Increase in number of active electrode tracks in organoid with microglia (24 out of 64) compared to organoids without microglia (1 out of 64) under basal conditions. Spontaneous bursting activity recorded in organoids with microglia.
Popova et al., 2021	Cortical organoid with microglia	DMEM-F12/Neurobasal	Excitatory, inhibitory (few), astrocytes, microglia	Wholemount							5 weeks after transplantation of microglia (20-week old organoids).	Increased synchronization and burst frequency in organoids with microglia compared to those without.

Whilst neurons derived from 3D brain organoids can be dissociated and further cultivated for single-cell patch-clamp recordings, organoids wholemounts or acute slices have the crucial advantage of retaining mostly intact tissue organization and neuronal circuits. For the purpose of this review, we will only discuss electrophysiological results obtained from recordings in either intact or sliced organoids.

The electrophysiological properties of cortical organoids were initially assessed using whole-cell patch-clamp techniques in human cortical spheroids (hCS), presenting both cortical excitatory neurons and astrocytes. A small current injection was sufficient to elicit action potentials (APs) in 80% of the recorded cortical excitatory neurons at day 90–130. Moreover, it was possible to record spontaneous excitatory postsynaptic currents (sEPSCs) in 86% of patched neurons, indicating the formation of functional excitatory neural networks (Pasça et al., 2015). However, no data on capacitance or input resistance for these recorded neurons was reported, rendering it difficult to make any assumption on their maturity.

An important feature of dorsal forebrain organoids is the spontaneous development of astrocytes, as observed during normal neurodevelopment. Astrocytes arise at late stages of cortical development, around gestational week (GW) 18 in humans, from the same radial glial precursors as excitatory cortical neurons, and after most of cortical neurogenesis has already occurred (Cadwell et al., 2019). Astrocytes play key roles in synaptic transmission, notably neurotransmitter recycling and K^+^ buffering. Additionally, they play active roles in synaptic development, especially control of synapse formation, function and pruning (Allen et al., 2012; Freeman and Rowitch, 2013). Astrocytes generated in the hCS model were found to closely resemble primary *in vivo* human astrocytes in terms of their transcriptional signatures. Moreover, they were functionally able to fulfill the same roles of astrocytes *in vivo*, including glutamate uptake *via* specific excitatory amino acid transporters, phagocytosis of synaptosomes and induction of synapse formation, important for cortical development and proper network activity (Sloan et al., 2017).

hCSs have been used to model Angelman Syndrome (AS), a neurodevelopmental disease associated with epilepsy (Sun et al., 2019). In control hCS, a maximum spike frequency of 18 Hz at day 120–150 was recorded. Maximum spike frequency was significantly increased in AS-derived spheroids and this hyperexcitability was found to be due to an increase in the expression of BK channels that in turn increases the size of the fast after-hyperpolarization (Sun et al., 2019). This finding provided unprecedented mechanistic insights into how a mutation in the ubiquitin protein ligase E3A (UBE3A) can result in seizures observed in AS. Importantly, this result was also recapitulated in an AS animal model (Sun et al., 2019). To be noted, a higher-order network activity analysis remains unexplored in hCS models, and as such, the full maturation of cortical circuits has not been assessed.

Network activity has been investigated in a different cortical organoid model (Trujillo et al., 2019). Cortical organoids at 180 days of differentiation showed TTX-sensitive trains of APs, which due to the late stage of maturation required a higher amount of current to be elicited. Spontaneous AP generation comparable to mature rodent excitatory pyramidal cells (around 15 Hz) was recorded. sEPSCs, blocked by NBQX and AP-5 were also present. Weekly extracellular recordings of spontaneous electrical activity using MEA plates containing 64 low-impedance electrodes and a total of 512 channels was also used to assess network activity. Using this method, high spike frequency (around 18 Hz) after 10 months in culture (Trujillo et al., 2019) were also recorded. Whilst this appears to be the same level of excitability recorded in the hCS model after 4–5 months in culture, differences in analysis hamper proper comparison (Sun et al., 2019; Trujillo et al., 2019). Over the course of 10 months, cortical organoids exhibited a steady increase in firing rate, burst frequency, synchronicity and population spiking. This cortical organoid model was used to investigate changes in network activity in organoids derived from patients with CDLK5-deficiency disorder (CDD), a neurodevelopmental disorder associated with early-onset epilepsy (Olson et al., 2019; Negraes et al., 2021). MEA recordings showed consistent increases in electrical activity in CDD-derived cortical organoids compared to controls, as well as a significant increase in mean firing rate and network synchronicity early in development (Negraes et al., 2021).

Another feature of this model is the robust oscillatory activity, which can be observed after 6 months of differentiation (Trujillo et al., 2019). Interestingly, the onset of oscillations is correlated with the emergence of a small population of GABAergic interneurons (10–15% of total neurons) indicating that GABAergic transmission is necessary for the maintenance of oscillatory activity. Eventually, the oscillatory activity transitions to more irregular patterns, and synchronous network events observed in the organoids at late stages share some similar features with those extracted from preterm human EEG data (Trujillo et al., 2019). Although in lesser numbers, GABAergic neurons have also been reported in the hCS model (Sun et al., 2019; Trujillo et al., 2019). It is still unclear how GABAergic interneurons, which originate from the ganglionic eminences of the ventral forebrain, can develop in organoid models specified to the dorsal forebrain (Wonders and Anderson, 2006). A proliferative niche for cortical interneurons (cINs) in the developing cortex of humans and primates, but not in mice, has been previously reported (Letinic et al., 2002; Radonjic et al., 2014). However, subsequent studies have suggested that this finding may be due to the use of markers that are not specific for the GABAergic lineage in humans, rather than a novel human-specific proliferative zone (Ma et al., 2013). Yet, a very recent paper has shown that some human cortical progenitors derived from fetal samples can generate both excitatory and inhibitory neurons, suggesting that the presence of a limited number of GABAergic neurons in dorsal forebrain organoids may be due to a human-specific developmental process (Delgado et al., 2021).

## Assembloids Recapitulate Cortical Cellular Diversity and Inter-Regional Connectivity

### Cortico-Subpallial Assembloids

Cortical interneurons (cINs) originate from progenitors located in the ganglionic eminences (GEs) of the ventral forebrain. GEs, also known as the subpallium, comprises three divisions: the lateral (LGE), medial (MGE) and caudal (CGE) ganglionic eminences. Around GW15 in humans, cINs generated in the subpallium enter the dorsal forebrain *via* tangential migration and disperse throughout the developing cortex (Ma et al., 2013). This migratory process is exceptionally protracted in humans through the second year of life (Paredes et al., 2016). Once they reached the cortex, interneurons undergo an activity-dependent maturation process and integrate into neural circuits where they play a key role in the generation and maintenance of the excitation/inhibition balance of the cortex (Kepecs and Fishell, 2014).

**FIGURE 1:**
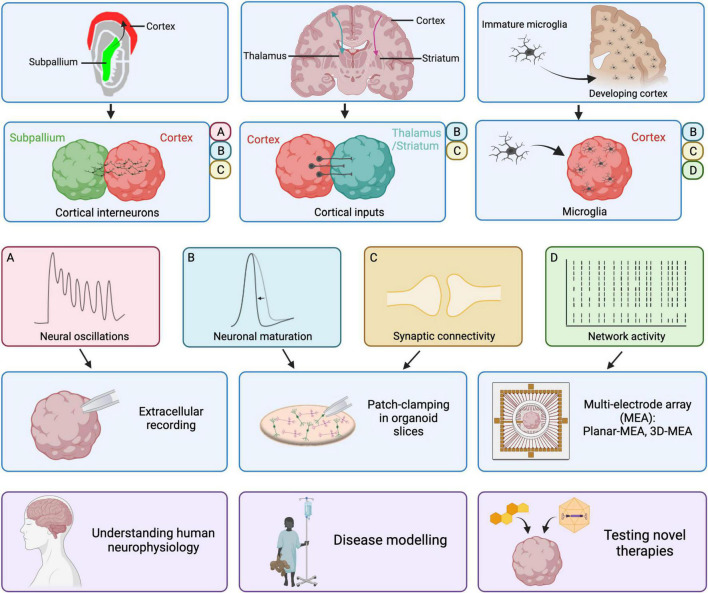
Improvements in cortical organoid models and their electrophysiological properties. Recent improvements in cortical organoid models include the inclusion of cortical interneurons with cortico-subpallial assembloids, the reproduction of cortical inputs to thalamus or striatum in cortico-thalamic and cortico-striatal assembloids and the addition of microglia derived from fetal samples or iPSCs. The presence of cortical interneurons in cortico-subpallial assembloids results in the emergence of neural oscillations observed *via* extracellular recordings of local-field potentials. Moreover, patch-clamping in slices revealed that migrated interneurons mature on the cortical side and form functional synapses. In cortico-thalamic and cortico-striatal assembloids, cortical neurons form functional synapses onto target thalamic or striatal neurons. Those cortical inputs result in increased neuronal maturation, associated with a shorter AP half-width, in thalamic or striatal neurons. The addition of microglia onto cortical organoids or cerebral organoids results in increased network activity as recorded using multi-electrode arrays. Moreover, the presence of microglia led to an increase in sodium and potassium currents in recorded neurons, indicative of maturation, as well as increased frequency of spontaneous synaptic activity. Ultimately, progress in the electrophysiological characterization and maturation of cortical organoids will allow us to improve our understanding of human neurophysiology, generate better disease models and test novel therapies more reliably. Created with BioRender.com.

This migratory process was recapitulated in assembloid models through fusion of two organoids: one specified to the cortex and one to the subpallium (Figure 1; Bagley et al., 2017; Birey et al., 2017; Samarasinghe et al., 2021). In this cortico-subpallial assembloid model, subpallium-derived cINs migrate into the cortical side, change their morphology and form synapses with glutamatergic neurons. Subpallium-derived cINs undergo tangential migration in a saltatory manner, a defining characteristic of interneuron migration *in vivo* (Guo and Anton, 2014). Spontaneous inhibitory postsynaptic currents (sIPSCs) and sEPSCs were recorded in both inhibitory and excitatory neurons in the cortical side after interneuron migration, indicating that cINs integrate into the cortical network (Birey et al., 2017). Importantly, migrated interneurons show increased AP firing compared to both non-migrated interneurons and interneurons in unfused subpallial spheroids (Birey et al., 2017). This aligns with the activity-dependent maturation process that interneurons undergo following their integration in excitatory neuronal networks, which has also been observed in the developing cortex *in vivo* (Kepecs and Fishell, 2014).

Network activity has also been assessed using extracellular local field potential recordings in cortico-subpallial assembloid models (Samarasinghe et al., 2021). The authors showed robust oscillatory activity over a 5-min period in assembloids after 100 days in culture. Spectral density analysis revealed multiple oscillatory peaks, ranging from 1–100 Hz. Interestingly, there was no measurable oscillatory activity in cortex-cortex fusions, indicating that the presence of cINs is required for the generation of higher-order network activities such as multifrequency oscillations (Samarasinghe et al., 2021). This is in accordance with the emergence of complex oscillatory activity observed after the detection of GABAergic interneurons in cortical and cerebral organoid models (Trujillo et al., 2019; Fair et al., 2020), as well as the evidence of the regulatory role of interneurons in the oscillatory processes in the rodent cortex (Kalemaki et al., 2018).

### Cortico-Striatal Assembloids

Cortico-striatal projections are essential components of the forebrain circuitry that coordinate motivated behavior and movement (Shepherd, 2013). They are unidirectional neuronal projections: the cortex communicates monosynaptically to the striatum, while the striatum communicates only indirectly with the cortex *via* downstream circuits. Two distinct classes of excitatory cortical pyramidal neurons project to the striatum: intratelencephalic (IT) and pyramidal tract (PT) neurons (Shepherd, 2013). Cortical neurons projecting to the striatum primarily connect to GABAergic medium spiny neurons. Dysfunctions in the cortico-striatal pathway are thought be involved in several neurodevelopmental diseases including autism spectrum disorder, schizophrenia and obsessive-compulsive disorder.

Specific differentiation patterning has been developed for the derivation of lateral ganglionic eminence (LGE), from where striatal GABAergic neurons originate, and generation of human-derived striatal spheroids (hStrS). hStrS present mostly GABAergic neurons (57%), with a small group of glutamatergic neurons, astrocytes and oligodendrocytes (Miura et al., 2020). GABAergic neurons in hStrS show complex arborization with dendritic spines harboring thick spine heads, reminiscent of the morphology of developing medium spiny neurons *in vivo*. hStrS neurons displayed characteristic electrophysiological properties of striatal medium spiny neurons including inward rectification, slow-ramp depolarization and a hyperpolarized resting membrane potential (−78 mV), as observed in rodents’ medium spiny neurons postnatally (Peixoto et al., 2016). Fusion of hStrS to hCS lead to formation of cortico-striatal assembloids (Figure 1). Over 3 weeks, unilateral projections from the hCS to the hStrS were formed, consistent with the organization of cortico-striatal circuits *in vivo*. Importantly, both IT and PT neurons were identified amongst the cortical neurons projecting to the hStrS, thus representing the diversity of cortico-striatal projections observed *in vivo*. Optogenetic stimulation of cortical neurons resulted in evoked EPSCs recorded in hStrS, showing that projecting cortical neurons form functional synaptic connections with striatal neurons. Crucially, neurons in the hStrS show increased excitability and a higher frequency of sEPSCs when fused to hCS compared to unfused (Miura et al., 2020). Moreover, individual APs had shorter half-widths, indicating a more mature phenotype. This confirms the importance of glutamatergic projections from the cerebral cortex for proper maturation of striatal medium spiny neurons.

### Cortico-Thalamic Assembloids

Reciprocal cortico-thalamic projections are established during development and are critically important for sensory-motor processing, attention and arousal (Lopez-Bendito and Molnar, 2003). Thalamic dysfunction has been implicated in a range of neurological disorders such as ASD, schizophrenia and epilepsy. Recently, thalamic organoids principally containing excitatory cells of thalamic origin (GBX2 and OTX2-positive) have been generated (Xiang et al., 2019). Upon fusion with cortical organoids, both cortico-thalamic and thalamo-cortical connections were formed, recapitulating the reciprocal connections observed between the cortex and thalamic nuclei during development *in vivo* (Figure 1; Xiang et al., 2019; Fligor et al., 2021). Electrophysiological characterization of thalamic neurons showed that around 50% of recorded cells fired APs both in fused and non-fused hThOs. However, a higher firing frequency could be observed in fused compared to unfused hThOs, suggesting that cortical inputs are involved in the functional maturation of thalamic neurons (Xiang et al., 2019). It was not reported whether the electrophysiological properties of cortical neurons are similarly impacted by thalamic inputs.

### Cortico-Motor Assembloids

In the cerebral cortex, pyramidal cells known as corticofugal glutamatergic neurons send long-range projections to the hindbrain and spinal cord in order to induce muscle contraction and motor movement (Lodato and Arlotta, 2015; Kiehn, 2016). This cortico-motor pathway is necessary for the generation of coordinated movement. Damage or degeneration of the cortico-motor circuit, which can for example be due to traumatic injury, amyotrophic lateral sclerosis (ALS) or auto-immune disease can thus result in severe motor impairments (Blesch and Tuszynski, 2009).

Recently, the generation of a spheroid specified to the hindbrain/cervical spinal cord (hSpS) has been reported, which contained neurons belonging to a diversity of spinal domains: dl1-6, V0, V1, V2a and V2b, but not V3 (Delile et al., 2019; Andersen et al., 2020). hSpS present with glutamatergic, GABAergic and cholinergic spinal motor neurons, as well as astrocytes and oligodendrocytes (Andersen et al., 2020). In order to verify functional maturation, hSpS were fused to a cortical spheroid (hCS) generating a cortico-spinal assembloid. Processes extending from the hCS into the hSpS were observed already 5 days after fusion. Projections from the hSpS to the hCS were also observed but to a lower extent. To probe whether hCS-derived projections formed functional connections with spinal neurons, hCS were transduced with an AAV construct containing the light-sensitive opsin Chrimson and voltage-clamp recordings in hSpS-derived spinal neurons were performed. Light-evoked stimulations could reliably induce post-synaptic currents in 2 out of 30 recorded hSpS-derived neurons and those responses were blocked by TTX, indicating that cortical neurons had formed functional synaptic connections with spinal neurons (Andersen et al., 2020). Skeletal muscle spheroids (hSkM) were also derived from iPSCs and fused to the hCS-hSpS assembloid. Remarkably, optogenetic stimulation in the hCS could reliably induce skeletal muscle contraction, but just in the presence of an hSpS in-between, indicating the formation of disynaptic connections (Andersen et al., 2020). The success rate for inducing those types of contractions was 81% at 5 weeks and dropped to 50% after 8 weeks in culture. However, assembloids at 8 weeks were more likely to sustain repeated contractions, suggesting a more robust response after long-term culture.

## Increasing Cortical Organoids Cellular Complexity: Potential Role of Microglia in Network Maturation

Microglia are the brain’s resident immune cells and play important roles in brain development, notably in synaptic maturation, transmission and pruning, thus shaping cortical networks (Stevens et al., 2007; Béchade et al., 2013; Schafer et al., 2013; Miyamoto et al., 2016; Mosser et al., 2017). Indeed, microglia support both synapse formation and elimination in an activity-dependent manner (Schafer et al., 2012; Parkhurst et al., 2013). Microglia also restrict the number of neuronal progenitors in the cortical SVZ and support the survival and maturation of cortical neurons (Cunningham et al., 2013; Ueno et al., 2013). Finally, they also play a role in the laminar positioning of cortical interneurons, thus impacting cortical wiring (Squarzoni et al., 2014).

While the cerebral cortex arises from the developing neuro-ectoderm, and cortical oragnoids are generated upon ectoderm patterning, microglia originate from the mesoderm. Due to the different germ layer origin, microglial cells are not present in cortical organoids and need to be infiltrated from external sources (Figure 1). Transplantation of primary human fetal microglia or iPSC-derived microglial progenitors (Fagerlund et al., 2021; Popova et al., 2021) showed that microglia or their precursors can spontaneously integrate in the organoid tissue, eventually transitioning from an immature amoeboid state to a ramified morphology indicating a homeostatic surveillance role (Fagerlund et al., 2021; Popova et al., 2021).

Cortical organoids at 15-weeks transplanted with human microglia purified from mid-gestation cortical fetal tissue showed increased synchronization and burst frequency of neural activity measured with MEAs (Popova et al., 2021). This suggests that microglia play a key role in the maturation of network activity in cortical organoids. Moreover, immunostaining of Vglut1 and PSD95 showed a significant reduction in synaptic puncta, and microglia showed integration of synaptic material, consistent with their role in synaptic pruning and circuit refinement (Popova et al., 2021). However, the number of microglia in the organoids drastically decreased 5 weeks after transplantation, hampering long term analysis of the role of microglia in network maturation.

Erythromyeloid progenitors (EMPs), microglia precursors, were generated from iPSCs and incorporated to cerebral organoids at an early maturation stage (Fagerlund et al., 2021). The incorporated EMPs infiltrated the organoid, establishing a microglial population, which then colonized the organoid. Differentiated microglia were found to interact with synapses, reducing the number of synaptic puncta, without inducing an inflammatory response. Interestingly, when recorded using whole-cell patch clamping between 107–165 days in culture, organoids containing microglia showed increases in both sodium and potassium current densities, without any changes in the basal electrophysiological parameters of the neurons (Fagerlund et al., 2021). This translated in an increased prevalence of neurons able to fire repetitive APs (Fagerlund et al., 2021). Importantly, in this model sEPSCs were recorded in cerebral organoids with microglia but not in those without. Finally, 3D-MEA recordings of organoid slices revealed that the presence of microglia robustly increases spontaneous network activity (Fagerlund et al., 2021). Microglia-containing slices exhibited varied activity patterns ranging from irregular firing to regular bursting. However, oscillatory activity was not observed in this model. This suggests that neurons in microglia-containing slices exhibit signs of single-cell bursting characteristics, without having yet developed network features of synchronized population activity. This may be due to the lack of GABAergic neurons in the cerebral organoids analyzed at this timepoint (Fair et al., 2020). Moreover, given the heterogenous nature of cerebral organoids, it is unclear if the recorded neurons were of true cortical identity. It would therefore be interesting to repeat such detailed electrophysiological characterization in cortical organoids/assembloids with or without microglia.

## Discussion

Cortical organoids exhibit some degree of functional maturation at the cellular and network-level. The recent development of assembloid models has allowed recapitulation of the integration of cortical interneurons and inter-regional connectivity between the cortex and other regions through long-range projections (Figure 1). On one hand, the presence of cortical interneurons is critically important in the emergence of complex oscillatory activity. On the other hand, functional long-range projections shape the maturation of neurons receiving cortical inputs. Finally, the transplantation of microglia onto organoids enhances neuronal maturation and boosts network formation.

Despite this, the lack of comprehensive and standardized electrophysiological characterization hampers the direct comparison of cortical organoid protocols and prevents a complete understanding of the levels of functional maturity that can be achieved in those models. Ideally, a standardized approach for both the cellular and network level should be applied to all existing and novel cortical organoid protocols and electrophysiological data should be deposited on an open-access repository. Moreover, analyzing electrophysiology within the context of single-cell transcriptomic data would allow us to better understand to which extent cell diversity and functionality can be recapitulated. Future developments such as improvements of *in vitro* culture conditions, microfluidic platforms and vascularization could all play a role in further improving the maturation of cortical organoids.

Cortical organoids have been used in high-throughput screening platforms using calcium imaging as read-out to test the effect of novel therapeutic compounds (Negraes et al., 2021). The use of both low- and high-throughput electrophysiological techniques, such as patch-clamping and MEAs respectively, will help to better characterize the effectiveness of novel treatments in normalizing pathological neuronal and network activity. In the long term, ensuring the physiological relevance of cortical organoids will be key for understanding normal physiological processes in the human brain as well as for the generation of reliable disease models that can be used to understand disease mechanisms and test novel treatments to accelerate clinical translation for many neurological diseases.

## Author Contributions

CZ wrote the original manuscript and generated the figure and table. MK, SB, and GL provided guidance and revisions on the original manuscript. SB and GL conceptualized the manuscript. All authors contributed to the article and approved the submitted version.

## Author Disclaimer

The views expressed are those of the authors and not necessarily those of the NHS, the NIHR, or the Department of Health.

## Conflict of Interest

The authors declare that the research was conducted in the absence of any commercial or financial relationships that could be construed as a potential conflict of interest.

## Publisher’s Note

All claims expressed in this article are solely those of the authors and do not necessarily represent those of their affiliated organizations, or those of the publisher, the editors and the reviewers. Any product that may be evaluated in this article, or claim that may be made by its manufacturer, is not guaranteed or endorsed by the publisher.
